# The Impact of Scarcity on Consumers’ Impulse Buying Based on the S-O-R Theory

**DOI:** 10.3389/fpsyg.2022.792419

**Published:** 2022-06-10

**Authors:** Jingjing Zhang, Nan Jiang, Jason James Turner, Saeed Pahlevan-Sharif

**Affiliations:** ^1^Taylor’s Business School, Taylor’s University, Subang Jaya, Malaysia; ^2^Business School, Asia Pacific University of Technology and Innovation, Kuala Lumpur, Malaysia

**Keywords:** scarcity, impulse buying, fear of missing out, bandwagon effect, COVID-19

## Abstract

**Purpose:**

The global COVID-19 pandemic impacted the healthcare systems of every nation. The scarcity of medical protective equipment led to impulse buying at the early stages of the COVID-19 outbreak in China which resulted in stockpiling and the increase of prices by retailers and insufficiencies among frontline workers. This situation impacted epidemic control work and market order and is the context from which this paper identifies how the scarcity of medical protective equipment affected Chinese consumers’ impulse buying based on the theories of S-O-R model and bandwagon effect. The research provides insight into the mechanism of mediation (fear of missing out) and moderation (bandwagon) in the relationship between scarcity and impulse buying.

**Design/Methodology/Approach:**

This study uses convenience sampling, surveying 488 Chinese consumers through an online questionnaire. Smart-PLS was used to test the hypotheses.

**Findings:**

The empirical findings demonstrate that scarcity makes consumers fear missing the chance of getting protective medical equipment, leading ultimately to impulse buying. Besides, the scarcity effect on consumers’ impulse buying was found to depend on other consumers’ follow up behaviour in such emergency situations.

**Research Limitations/Implications:**

The findings provide managerial and theoretical insight and a point of reference for businesses in the implementation of a scarcity strategy. The findings will also prove useful to the Chinese Risk Response Department as it continuously improves its responses to the risk of consumers’ impulse buying during a pandemic.

**Originality/Value:**

This study consolidates and takes research forward in the areas of impulse buying and consumer behaviour, confirming the mediating effect of fear of missing out and the moderating effect of the bandwagon in the relationship between scarcity and impulse buying.

## Introduction

In January 2020, the World Health Organization (WHO) declared a global health emergency over a new virus, known as COVID-19. The ongoing spread of COVID-19 with a high risk globally (Du et al., 2020), impacted global healthcare systems and public health (Alves et al., 2020; Abbas, 2021), with citizens across the globe suffering from the symptoms of the virus and/or associated health concerns, such as psychology issues (Arafat et al., 2020). For China, which was the first country to be impacted by COVID-19 (Sun et al., 2021), the rapid spread of COVID-19 led to immediate pressure on the demand and supply of medical protective equipment (Zhang et al., 2021). Medical protective equipment became a scarce product, with no vaccines available for preventing COVID-19 at the early stages of the COVID-19 outbreak in China (Li M. et al., 2020). In short, China faced a significant challenge to source medical protective equipment (such as masks, alcohol, protective suits, disinfectants, medical gloves and goggles) to ensure protection against the virus.

The shortage of medical protective equipment brings a variety of issues which impact on the general public, one of which is the changes to consumer behaviour, such as impulse buying (Zhang et al., 2021) and/or panic buying (Arafat et al., 2020, 2021). Given the research focuses on impulse buying, it is important to provide a distinction between the two concepts to justify the focus and differentiate between impulse and panic buying. Impulse buying is defined as the spontaneous and immediate purchase of a product without any thoughtful and deliberate consideration of alternative or future implications (Pradhan, 2016; Moon et al., 2017; Cai et al., 2021; Rodrigues et al., 2021). Panic buying, in contrast, is defined as an occurrence involving negative feelings like fear, panic and feelings of uncertainty which influence behaviour and leads to people purchasing in higher quantities than they normally would without those negative feelings (Lins and Aquino, 2020), also known as stockpiling (Di Crosta et al., 2021; Taylor, 2021). On occasion, impulse buying has been referred to as unplanned buying or irrational behaviour (Lim and Yazdanifard, 2015; Ho and Lim, 2018; Li X. Y. et al., 2020; Islam et al., 2021) and is different from panic buying in that it can be seen as both rational (e.g., stockpiling essential goods that are in limited supply) and irrational (e.g., stockpiling non-essential products that are not in limited supply; Martin-Neuninger and Ruby, 2020). Panic buying is one of the antecedents of impulse buying behaviour, making impulse buying a more holistic concept and appropriate to use in the context of the COVID-19 pandemic (Ahmed et al., 2020; Primanto and Rahmawati, 2021).

Consumers’ impulsive consumption behaviour not only affects economic development (Lee and Yun, 2015; Wu and Li, 2018), it also significantly influences market order, with consumer impulse buying leading to stockpiling and the increase of prices by the seller (Cheriyan and Tamilarasi, 2020). One effective way to reduce the negative effect of impulse buying is to identify the impact of scarcity on impulse buying and underline the aims of this study, to identify how scarcity affects consumers’ impulse buying.

Previous research is comparatively limited in identifying the role of emotion in the relationship between scarcity and impulse buying during a pandemic and in the context of China which creates a gap for this research to examine. How consumers emotionally respond to an emergency as in this case, a global pandemic, is crucial to a better understanding of how consumers engage with the environment around them (Šrol et al., 2021). During this period, citizens of China were worried and anxious about the scarcity of medical protective equipment (Li X. Y. et al., 2020) which can lead to erratic or unconformist behaviours. Based on the S-O-R (stimulus, organism, response) model, environment stimulus can affect response through the emotion (organism; Mehrabian and Russell, 1974; Lee and Yun, 2015; Wu and Li, 2018; Yang et al., 2021). The scarcity of medical protective (stimulus) equipment may affect consumers’ impulse buying (response) through the consumers’ emotion (organism; e.g., fear of missing out), as the consumers’ fear of missing out is one type of emotion aroused by scarcity (Hodkinson, 2019) and regarded as one factor that can influence a consumers’ impulse buying (Casale and Flett, 2020). China had a huge demand for scarce medical protective products, and during the peak of the pandemic, existing supply could not match the daily demands of Chinese families. Consumers had to invest time and effort searching for those scarce medical protective products to avoid any chance of missing out on the chance to purchase products which could protect their family and as a result reduce the associated feelings of remorse (Mertens et al., 2020; Swennen et al., 2020).

Moreover, the bandwagon consumption that happened during the COVID-19 pandemic (Labad et al., 2021) resulted in consumers following each other to purchase masks and other medical protective equipment (Chen and Shi, 2020). This caused a waste of resources, affected the market and reduced the efficiency of resource allocation. The bandwagon effect contributed to impulse and panic buying and often resulted in stock outs during the pandemic (Yuen et al., 2020). Current research has explained that bandwagon consumption is always related to the scarcity of products (Ku et al., 2013; Sharma and Roy, 2016) and that the bandwagon effect has a significant impact on consumers’ impulse buying. This research will identify the role of the bandwagon in the relationship between scarcity and impulse buying during the pandemic which goes some way to addressing previously debated relationships in the context of consumer demand.

In summary, this study tries to verify the role of fear of missing out and bandwagon in the relationship between scarcity and impulse buying based on the theory of S-O-R and bandwagon effect. The findings may be helpful for identified stakeholders, such as retailers, consumers and the government, in responding to the risk of scarcity of medical protective equipment and consumers’ impulse buying.

## Theoretical Development and Hypotheses

### S-O-R Model

The S-O-R model asserts that stimuli from the environment affects an individuals’ affective (emotion) and cognitive (perception) reactions, which in turn influence individual behaviour (Mehrabian and Russell, 1974; Lee and Yun, 2015; Wu and Li, 2018). The stimulus is a trigger that arouses consumers and refers to marketing stimulus and/or situation stimulus (Chan et al., 2017; Kamboj et al., 2018). The organism is an internal state of an individual, which is represented by affective and cognitive states (Basha et al., 2022). It is also regarded as an intermediary state between the stimulus and response (Zheng et al., 2019). The last factor in the S-O-R model is the response which can also be called behaviour (Kamboj et al., 2018). The S-O-R model has been used to explain the relationship between scarcity and impulse buying (Chen and Yao, 2018; Islam et al., 2021; Zhang et al., 2021) and therefore deemed appropriate for this study to adopt and to guide the path of the environment (scarcity of medical protective equipment) on consumers’ impulse purchases. This study assumed that scarcity of medical protective equipment (environment) affects consumers’ impulse buying (response) through the mediating mechanism of fear of missing out (organism).

### Bandwagon Effect

The bandwagon effect refers to individuals doing certain things because others are doing them (Park et al., 2017; Mainolfi, 2020). In marketing, the bandwagon effect describes the phenomena of consumers following others’ behaviour and attitudes to purchase products and services (Tynan et al., 2010; Shayan et al., 2017) and is usually caused by demand-induced scarcity (Fu and Sim, 2011; Maxwell, 2014). This study focuses on the scarcity of medical protective equipment caused by the huge demand during the pandemic, which is also referred to as demand scarcity. In the case of scarce medical protective equipment, the bandwagon effect is an important factor worthy of attention.

**Figure 1:**
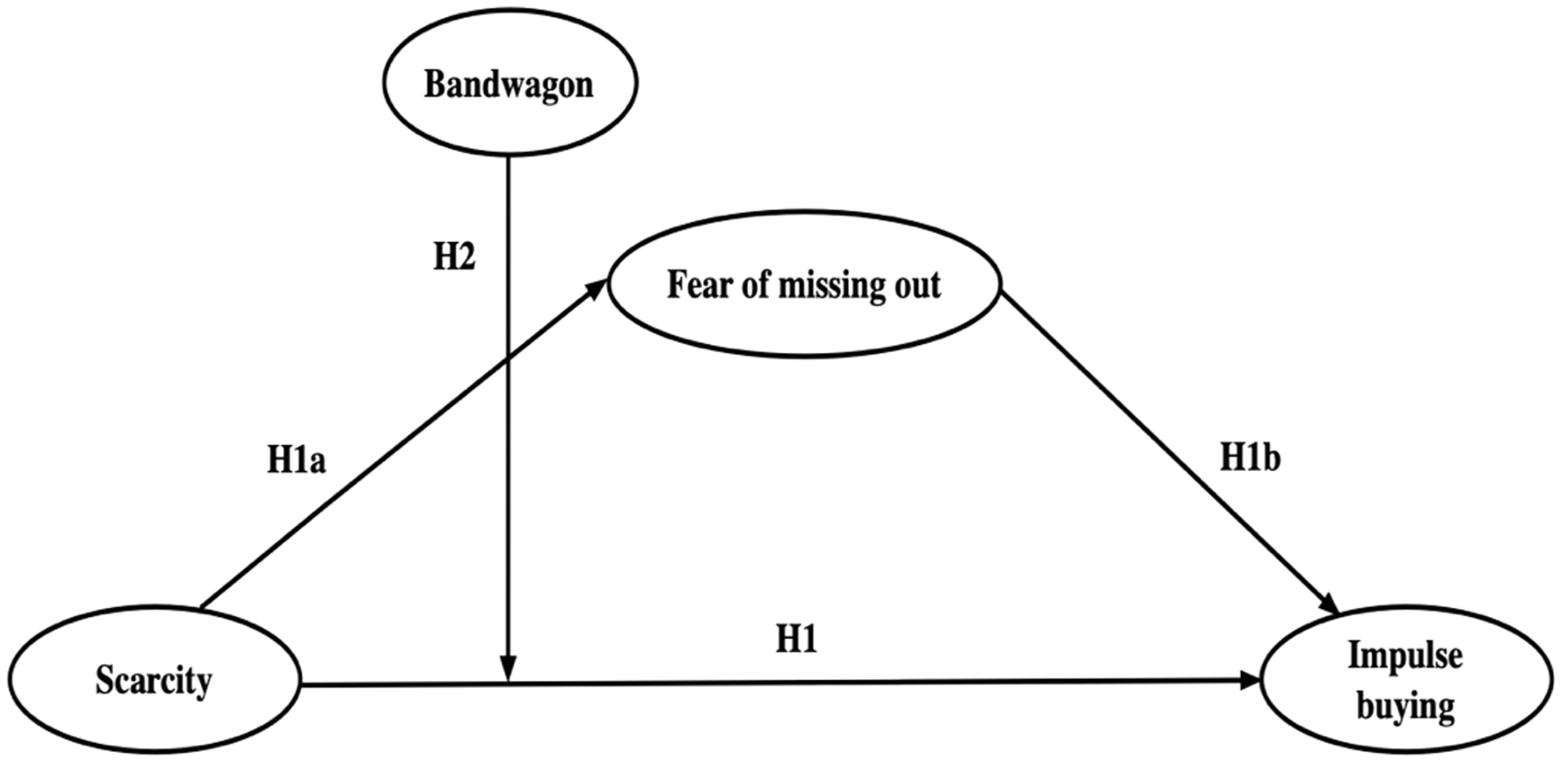
Research model.

As a result, this study constructs a framework which combines the theories of S-O-R and bandwagon, illustrated in Figure 1.

#### Relationship Between Scarcity, Fear of Missing Out and Impulse Buying

Generally, fear of missing out (FOMO) is defined as one type of emotion (Hayran et al., 2020; Zhang Z. et al., 2020), that describe consumer’s anxiety about missing the chance or experience that others have (Abel et al., 2016). In this study, FOMO is contextualised as the fear of missing the chance to purchase medical protective equipment. Consumers’ fear of missing out will induce impulse buying (Aydın, 2018) which has commonly been defined as the spontaneous, immediate purchase of the product without any thought or deliberate consideration of alternative or future implications (Pradhan, 2016; Cai et al., 2021; Rodrigues et al., 2021). It involves an investment made through a given motivation in which the decision to purchase something does not depend on profound thought processes. Impulse buying can be induced by external stimuli and personal emotions (Addo et al., 2020; Ahmed et al., 2020; Huang and Cai, 2021) with current research indicating that consumers’ perceived scarcity of products leads to a fear of ‘missing out’ and therefore influences a consumers’ choices and decisions (Hodkinson, 2019; Zhang Z. et al., 2020). Thus, this study focuses on the scarcity of medical protective equipment and based on the S-O-R model, considers if scarcity influences consumers’ impulse buying by the FOMO and therefore proposes hypothesis 1.

*H1*: Fear of missing out (FOMO) mediates the relationship between scarcity and impulse buying.

#### Relationship Between Scarcity, Bandwagon and Impulse Buying

The bandwagon effect is usually triggered by scarcity (Van Herpen et al., 2009; Ku et al., 2013), particularly demand scarcity, which is associated with the bandwagon effect (Ku et al., 2013). The bandwagon effect might lead to a demand acceleration phenomenon which would increase the level of product scarcity (Li et al., 2021). In other words, the bandwagon effect may have an impact on the scarcity effect (Gierl and Huettl, 2010; Sharma and Roy, 2016), while scarcity has an effect on impulse buying (Cook and Yurchisin, 2017; Gupta and Gentry, 2019). In the context of COVID-19 and the purchase of protective medical products, the study will examine the scarcity effect on impulse buying depending on the level of the bandwagon effect. As a result, the following hypothesis is proposed.

*H2*: Bandwagon moderates the effect of scarcity on impulse buying.

## Research Methodology

### Research Design and Participant

This study used correlational cross-sectional, questionnaire-based research design to investigate the effect of medical protective products scarcity on Chinese consumers’ impulse buying. The target sample was Chinese consumers in China using convenience sampling given there was a strict lockdown in the country. The study constructed an electronic questionnaire using a professional online survey platform called SoJump with links to the questionnaire along with explanatory text disseminated across two social media channels, Wechat and QQ, which generated 488 valid responses (see Table 1). The questionnaire was written in Chinese and the respondents were voluntary participants in the study. Prior to carrying out the questionnaire ethical approval was given and a pilot study conducted to test for respondent understanding which revealed no misleading questions or redundancy. According to Hair et al. (2017, p. 24), an appropriate sample size should equal to ‘ten times the largest number of structural paths directed at a particular construct in the structural model’. In the context of this research using this justification, 44 responses would represent the smallest sample size, however, such a small sample size would not be representative and therefore, based on previous empirical studies in the area, a sample size around 300 responses was considered more suitable (Schoenherr et al., 2015; Rahi, 2017). Having distributed to Chinese consumers, the study received 488 responses which were deemed acceptable.

**Table 1:** Demographic profile of respondents.

**Characteristics**		**Frequency (n)**	**Percentage (%)**
**Gender**	Male	212	43.4
	Female	276	56.5
**Age**	25 and under	123	25.2
	26–35	215	44.1
	36–45	92	18.9
	46–55	43	8.8
	56 over	15	3.1
**Occupation**	Public official	226	46.3
	Unemployed	40	8.2
	Retired	12	2.5
	Student	92	18.9
	Other	118	24.2
**Income (RMB)**	2500 or less	146	29.9
	2501–3500	110	22.5
	3501–4500	90	18.4
	4501–5500	71	14.5
	5501–6500	31	6.4
	6501 and above	40	8.2
**Education Level**	High school (including Technical secondary school) and lower	96	19.7
	College degree	164	33.6
	Graduate degree	185	37.9
	Postgraduate degree or higher	43	8.8
**Medical protective products are scarcity**	Yes	352	72.1
	No	136	27.9
**Total**		488	100

### Measures

Creswell (2008) states that using existing validated instruments enables constructs to be measured for validity, reliability and accuracy. In this study, all the questions refer to the situation around the COVID-19 outbreak in China. This is context modification to the questions which have been adapted from previous research, with the reliability of the original scale considered good and above 0.7. In addition, 50 samples were pre-tested, in order to know the comprehensibility and effectiveness of the measurement items. All the values of Cronbach alphas (CA) are over 0.7, as seen in Table 2. A seven-point Likert scale was used for all items (where 1 = strongly disagree and 7 = strongly agree), as the seven-point scale has been widely used in marketing research (Cacciolatti and Lee, 2016; Joensuu-Salo et al., 2018 and Firman et al., 2020) and compared to the five-point Likert scale, the seven-point Likert scale provides more varieties of options thereby increasing the variability and statistical significance of the results.

**Table 2:** Results of the pilot test.

Constructs	Cronbach’s Alpha
Bandwagon	0.887
Fear of missing out	0.897
Impulse buying	0.882
Scarcity	0.885

#### Scarcity

To measure scarcity of medical protective products, this study adapted a 5-item scale by Wu et al. (2012). The reliability of the original scale was good as it was above 0.7. Respondents were asked to indicate levels of agreement towards each of the five statements on scarcity of medical protective products (e.g., ‘I think that the current supply of this bag is small’ modified to ‘I think that the current supply of medical protective products is small’).

#### Bandwagon

To measure consumers’ bandwagon effect on medical protective products, this study adapted a 4-item scale developed by Mainolfi (2020). The reliability of the original scale was good as it was above 0.7. Respondents were asked to indicate levels of agreement towards each of the four statements on the bandwagon effect associated with the purchase of medical protective products (e.g., ‘I only choose luxury brands that others buy’ modified to ‘I only choose the medical protective products that others buy’).

#### Fear of Missing Out

A 5-item scale was adapted to measure consumers’ fear of missing out on medical protective products (Kaur, 2020), the reliability of the original scale was good as it was above 0.7. Respondents were asked to indicate levels of agreement towards each of the five statements on the fear of missing out associated with the purchase of medical protective products (e.g., ‘Worried when others buy’ modified to ‘Worried when others buy the medical protective products’).

#### Impulse Buying

To measure consumers’ impulse buying of medical protective products, this study used 3-item scale developed by Darrat et al. (2016). The reliability of the original scale was good as it was above 0.7. Respondents were asked to indicate levels of agreement towards each of the three statements on how impulse buying is associated with the purchase of medical protective products (e.g., ‘I often buy things without thinking’ modified to ‘I often buy medical protective products without thinking’).

### Data Analysis

This study used structural equation modelling (SEM) to evaluate the fit of the research model and test the validity of the hypotheses, as SEM can assess the significance of moderators and mediators together expressed in a single equation, unlike other multiple regression analysis (Hair et al., 2014). PLS-SEM tends to produce greater statistical power, which means that PLS-SEM is more likely to generate a specific relationship significant when it is, in fact, significant in the population (Hair et al., 2017). Smart-PLS 3.3.2 was employed to examine the quality of the measurement model and test the relationships in the structural model. The bootstrapping method with 5,000 samples was used to test the hypotheses of this study to produce a more compelling and accurate analysis (Rasoolimanesh et al., 2021).

## Results

### Measurement Model

The measurement model is used to evaluate the relationships between the indicator variables and their corresponding construct(s). It determines which indicators to use for construct measurement and the directional relationship between construct and indicators (Hair et al., 2014, 2017). In general, it includes the test of composite reliability, average variance extracted (AVE) and discriminant validity (Hair et al., 2017). According to Hair et al. (2010) and Hair et al. (2014), the value of Cronbach’s alpha (CA) and composite reliability (CR) should be above 0.70, and each construct’s AVE be 0.50 or higher. Moreover, the achievement of discriminant validity (Fornell–Larcker criterion) is that the indicator loading is more significant than its cross-loading. Table 3 shows that the values of Cronbach’s alpha, composite reliability and AVE are all over the advised thresholds. The square root of AVE of each construct is also higher than its correlations with other constructs. This proves that discriminant validity is achieved (Table 4). The results are considered a satisfactory fit for the measurement model.

**Table 3:** Measurement model results.

Constructs	Items	Outer loadings	Cronbach’s alpha	Composite reliability	AVE
Bandwagon			0.880	0.918	0.736
	I buy medical protective products to be integrated into the social group I aspire to	0.837			
	I only choose the medical protective products that others buy	0.875			
	I like owning the medical protective products worn by others	0.836			
	I buy very popular products	0.882			
FOMO			0.909	0.932	0.733
	I am anxious when missing the chance to get the medical protective products	0.846			
	Keep tabs on others	0.880			
	Worried when others buy the medical protective products	0.842			
	Follow others’ shopping pattern	0.842			
Impulse buying			0.875	0.923	0.800
	‘Just do it’ describes the way I buy things	0.871			
	I often buy medical protective products without thinking	0.906			
	‘I see it, I buy it’ describes me	0.906			
Scarcity			0.880	0.913	0.677
	I think that the current supply of medical protective products is small	0.822			
	I think the medical protective products is selling out soon	0.844			
	I think that many people will buy medical protective products	0.848			
	I feel that the shortage of medical protective products will cause many people to buy	0.763			
	I think the supplies only limit the number of masks for each person and will cause a lot of people to buy	0.834			

**Table 4:** Discriminant validity assessment using Fornell–Larcker criterion.

Construct	(1)	(2)	(3)	(4)
**(1)** Bandwagon	0.858			
**(2)** FOMO	0.563	0.856		
**(3)** Impulse buying	0.570	0.516	0.894	
**(4)** Scarcity	0.517	0.488	0.485	0.823

### Structural Model

The results are shown in Table 5 support all the hypotheses presented in the model, as the value of *p* is less the 0.05 with a *t*-value below 1.96 (Hair et al., 2017). Table 5 demonstrates the mediation of fear of missing out and moderation of bandwagon on the relationship between scarcity and impulse buying. Firstly, it presents the indirect effect between scarcity and impulse buying through FOMO was significant (*β* = 0.095, *t* = 3.446, *p* < 0.01). That is, FOMO positively mediates the relationship between scarce medical protective equipment and Chinese consumers’ impulse buying.

**Table 5:** Path coefficients.

Path	Std. Beta ( *β*)	*T*-value	*p*-Value	Hypotheses	Decision
Scarcity → FOMO→ Impulse buying	0.095	3.446	0.001^**^	H1	Supported
Scarcity → impulse buying	0.233	4.428	0.000^***^		
Scarcity → FOMO	0.488	11.986	0.000^***^		
FOMO → Impulse buying	0.194	3.692	0.000^***^		
Moderating Effect → Impulse buying	0.099	2.894	0.004^**^	H2	Supported

* *p* < 0.05;

** *p* < 0.01; ^***^p < 0.001.

**Figure 2:**
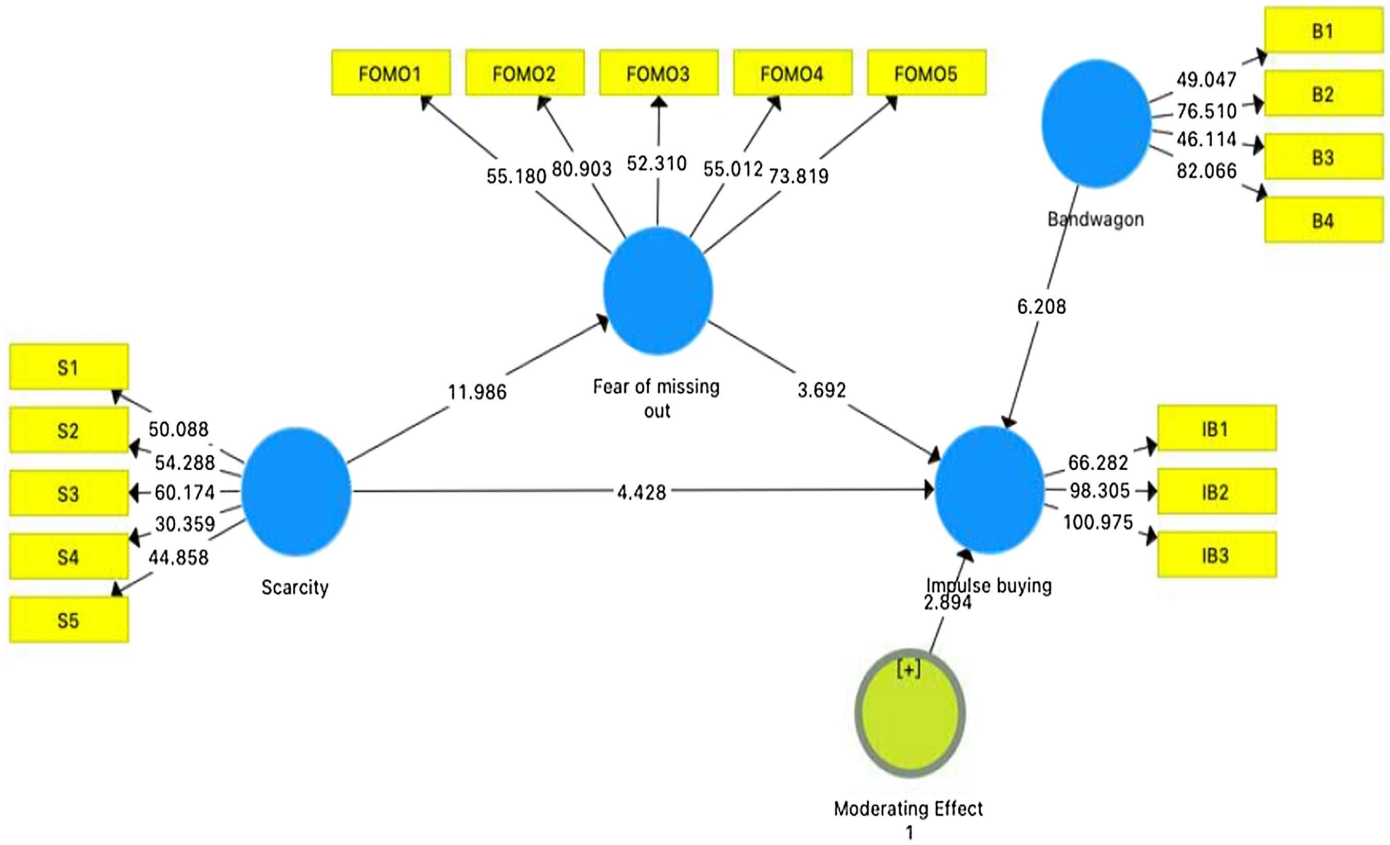
The results of the structural model.

Secondly, the results show the moderation of the bandwagon on the relationship between scarcity and impulse buying (*β* = 0.099, *t* = 2.894, *p* < 0.001). It means that increasing the bandwagon effect will enhance the scarcity effect, which may make consumers more impulsive. In short, H1 and H2 are supported. Figure 2 depicts the results of the structural model.

## Discussion

Fear of missing out was found to play a mediating role in the relationship between scarcity and impulse buying. In other words, the high level of scarcity associated to medical protective equipment increased consumers’ fear of missing out on the products, making them more impulsive in their purchasing behaviour. This finding supports the S-O-R model, which contends that the stimuli from the environment affects an individual’s emotion which in turn influences individual behaviour (Wu and Li, 2018). Zhang W. et al. (2020b) provide further corroborated, arguing that FOMO mediates the relationship between consumers’ perceived scarcity and choice. This finding provides insight into a customer’s emotion and its importance to consumer behaviour. Retailers not only confined to the medical sector should consider this when they wish to use external or internal environmental factors to attract consumers and influence purchase intentions.

The study indicated that the bandwagon effect moderates the relationship between scarcity and impulse buying. It revealed that the impact of scarcity of medical protective equipment on consumers’ impulse buying depended on the level of the bandwagon effect. When consumers found others purchasing medical protective equipment, their perception of the products’ scarcity engendered impulse behaviour. This finding is supported by previous studies (Gierl and Huettl, 2010; Sharma and Roy, 2016) which concluded that the bandwagon effect positively impacted the scarcity effect. This implies that retailers and/or the government, individually and collectively, may wish to reduce the scarcity effect by implementing control levers over the bandwagon effect which can reduce the impact which has been observed previously in other retail sectors and in the context of seasonality.

## Conclusion

### Theoretical Implications

Based on the findings, this study reveals several theoretical implications. First, the results extend existing research on scarcity, impulse buying, fear of missing out and the bandwagon effect. Second, this study underlines the argument that the combined theories of S-O-R and bandwagon effect are able to explain the phenomenon of scarcity and its impact on consumers’ impulse buying. Finally, this study provides additional insight into the relationship between scarcity and impulse buying, taking into account the underlying mediating (fear of missing out) and moderating (bandwagon) variables. Using the model generated in this study, consumers’ reactions (impulse buying) to scarce products can be better explained.

### Practical Implications

This study also reveals several practical implications. First, the results illustrate that the scarcity effect on consumers’ impulse buying increases with the fear of missing out, which can assist retailers in developing mediating mechanisms and scarcity strategies to maximise the possible effect of scarcity on consumers’ purchase intention. Second, the results encourage retailers to pay attention to the bandwagon effect, as a high level of bandwagon effect may increase the scarcity effect on consumers’ impulse buying. Retailers may incorporate this into their scarcity strategy, creating ‘bandwagon consumption’ to attract more potential consumers. Third, the results of this research provide useful information for the Chinese Crisis Response Department so that they may take proactive measures to reduce the crisis brought about by consumers’ impulse buying during the pandemic. The Crisis Response Department may consider taking preventive measures for consumers’ reactionary behaviour (bandwagon) to curtail the effect of scarcity, reducing consumers’ impulse buying and better controlling purchase behaviour during a pandemic or state of emergency. For instance, the Crisis Response Department could inform and educate the general public that bandwagon consumption could lead to insufficient protective equipment being available for frontline staff and likely cause prices to soar, underlining the necessity to limit the purchase quantity of medical protective equipment. Finally, this study, through its analysis of the scarcity effect on consumers’ impulse buying, can assist consumers realise that scarcity is employed as a marketing tactic to promote irrational purchase behaviour. The findings from the research can make customers more aware of their emotions and the role they play in the purchasing of products and services which in turn can save them money as scarce products are generally higher priced (Van Herpen et al., 2009).

## Limitations and Future Research

The study does have its limitations, which do not impact on the validity of the research but are nevertheless considered limitations and linked to further recommendations. First, this study only investigated the fear of missing out without considering other potential mediators. It is surmised that with the underlying S-O-R model, there could be other types of affection that can serve as mediators. Future research may prefer identifying additional mediating variables which belong to emotion and cognition (e.g., fear and judgment). Second, this study attempted to understand the scarcity effect on impulse buying with the help of two theories: S-O-R and bandwagon effect. Although the research model is comprehensive with the rationale for selection compelling, there are other potential theories which could have been used to understand the same effect. For further research, other theories could be employed, such as the competitive arousal model and theory of fear, to examine consumers’ impulse buying. Finally, this study was cross-sectional in nature. The data was collected at one specific time in China (January–March 2020). Further research could be extended, combining both quantitative and qualitative approaches, to gain insight into the long-term impact and behaviour of COVID-19 on the purchase behaviour of medical protective equipment.

## Data Availability Statement

The raw data supporting the conclusions of this article will be made available by the authors, without undue reservation.

## Ethics Statement

The studies involving human participants were reviewed and approved by Human Ethics Committee, Taylor’s University. Written informed consent for participation was not required for this study in accordance with the national legislation and the institutional requirements.

## Author Contributions

JZ, NJ, and JT: conceptualization, writing—review and editing and visualization. JZ: methodology, software, formal analysis, investigation, resources, data curation and writing—original draft preparation. JZ, NJ, JT, and SP-S: validation. NJ, JT, and SP-S: supervision. All authors contributed to the article and approved the submitted version.

## Conflict of Interest

The authors declare that the research was conducted in the absence of any commercial or financial relationships that could be construed as a potential conflict of interest.

## Publisher’s Note

All claims expressed in this article are solely those of the authors and do not necessarily represent those of their affiliated organizations, or those of the publisher, the editors and the reviewers. Any product that may be evaluated in this article, or claim that may be made by its manufacturer, is not guaranteed or endorsed by the publisher.

## References

[ref1] Abbas J. (2021). Crisis management, transnational healthcare challenges and opportunities: The intersection of COVID-19 pandemic and global mental health. Res. Global. 3:100037. doi: 10.1016/j.resglo.2021.100037

[ref2] Abel J. P. Buff C. L. Burr S. A. (2016). Social media and the fear of missing out: scale development and assessment. J. Bus. Eco. Res. 14, 33–44. doi: 10.19030/jber.v14i1.9554

[ref500] Addo P. C. Jiaming F. Kulbo N. B. Liangqiang L. (2020). COVID-19: fear appeal favoring purchase behavior towards personal protective equipment. The Service Industries Journal 40, 471–490. doi: 10.1080/02642069.2020.1751823, PMID: 32668635

[ref3] Ahmed R. R. Streimikiene D. Rolle J. A. Pham A. D. (2020). The COVID-19 pandemic and the antecedants for the impulse buying behavior of US citizens. J. Comp. 12, 5–27. doi: 10.7441/joc.2020.03.01

[ref4] Alves J. C. Lok T. C. Luo Y. Hao W. (2020). Crisis management for small business during the COVID-19 outbreak: survival, resilience and renewal strategies of firms in Macau. Res. Square. 1–29. doi: 10.21203/rs.3.Rs-34541/v1

[ref5] Arafat S. M. Kar S. K. Kabir R. (2021). Possible controlling measures of panic buying during COVID-19. Int. J. Ment. Heal. Addict. 19, 2289–2291. doi: 10.1007/s11469-020-00320-1, PMID: 32837417 PMC7241580

[ref6] Arafat S. Y. Kar S. K. Marthoenis M. Sharma P. Apu E. H. Kabir R. (2020). Psychological underpinning of panic buying during pandemic (COVID-19). Psychiatry Res. 289:113061. doi: 10.1016/j.psychres.2020.113061, PMID: 32413711 PMC7202808

[ref7] Aydın H. (2018). Explaining the effects of fear of missing the developments in social media (Fomo) on instinctive purchases by self-determination theory. Int. J. Eco. Admin. Stu. 17, 415–426. doi: 10.18092/ulikidince.439179

[ref8] Basha N. K. Aw E. C. X. Chuah S. H. W. (2022). Are we so over smartwatches? Or can technology, fashion, and psychographic attributes sustain smartwatch usage? Technol. Soc. 69:101952. doi: 10.1016/j.techsoc.2022.101952

[ref9] Cacciolatti L. Lee S. H. (2016). Revisiting the relationship between marketing capabilities and firm performance: The moderating role of market orientation, marketing strategy and organisational power. J. Bus. Res. 69, 5597–5610. doi: 10.1016/j.jbusres.2016.03.067

[ref10] Cai Z. Gui Y. Wang D. Yang H. Mao P. Wang Z. (2021). Body image dissatisfaction and impulse buying: A moderated mediation model. Front. Psychol. 12:653559. doi: 10.3389/fpsyg.2021.653559, PMID: 33981278 PMC8107384

[ref11] Casale S. Flett G. L. (2020). Interpersonally-based fears during the COVID-19 pandemic: reflections on the fear of missing out and the fear of not mattering constructs. Clin. Neuropsychiatry 17, 88–93. doi: 10.36131/CN20200211, PMID: 34908975 PMC8629079

[ref12] Chan T. K. Cheung C. M. Lee Z. W. (2017). The state of online impulse-buying research: A literature analysis. Inf. Manag. 54, 204–217. doi: 10.1016/j.im.2016.06.001

[ref13] Chen A. Shi Y. (2020). The impact of risk appetite on public purchasing behavior: taking the purchase of masks as an example. Sci. Technol. Rev. 38, 86–92. doi: 10.3981/j.issn.1000-7857.2020.04.011

[ref14] Chen C. C. Yao J. Y. (2018). What drives impulse buying behaviors in a mobile auction? The perspective of the stimulus-organism-response model. Telematics Inform. 35, 1249–1262. doi: 10.1016/j.tele.2018.02.007

[ref15] Cheriyan A. Tamilarasi D. S. (2020). Impulse buying during the pandemic times, with special reference of Kerala. PalArch’s J. Archaeol. Egypt/Egyptol. 17, 2757–2766.

[ref16] Cook S. C. Yurchisin J. (2017). Fast fashion environments: consumer’s heaven or retailer’s nightmare? Int. J. Retail Distrib. Manag. 45, 143–157. doi: 10.1108/IJRDM-03-2016-0027

[ref17] Creswell J. W. (2008). Educational Research: Planning, Conducting, and Evaluating Quantitative and Qualitative Research. 3rd Edn. Upper Saddle River. NJ: Pearson Education, Inc.

[ref18] Darrat A. A. Darrat M. A. Amyx D. (2016). How impulse buying influences compulsive buying: The central role of consumer anxiety and escapism. J. Retail. Consum. Serv. 31, 103–108. doi: 10.1016/j.jretconser.2016.03.009

[ref19] Di Crosta A. Ceccato I. Marchetti D. La Malva P. Maiella R. Cannito L.. (2021). Psychological factors and consumer behavior during the COVID-19 pandemic. PLoS One 16:e0256095. doi: 10.1371/journal.pone.0256095, PMID: 34398916 PMC8366984

[ref20] Firman A. Putra A. H. P. K. Mustapa Z. Ilyas G. B. Karim K. (2020). Re-conceptualization of business model for marketing nowadays: theory and implications. J. Asian Fin. Eco. Bus. 7, 279–291. doi: 10.13106/jafeb.2020.vol7.no7.279

[ref21] Fu W. W. Sim C. C. (2011). Aggregate bandwagon effect on online videos’ viewership: value uncertainty, popularity cues, and heuristics. J. Am. Soc. Inf. Sci. Technol. 62, 2382–2395. doi: 10.1002/asi.21641

[ref22] Gierl H. Huettl V. (2010). Are scarce products always more attractive? The interaction of different types of scarcity signals with products’ suitability for conspicuous consumption. Int. J. Res. Mark. 27, 225–235. doi: 10.1016/j.ijresmar.2010.02.002

[ref23] Gupta S. Gentry J. W. (2019). Should I buy, hoard, or hide? -consumers’ responses to perceived scarcity. Int. Rev. Retail Distrib. Consum. Res. 29, 178–197. doi: 10.1080/09593969.2018.1562955

[ref24] Hair J. Sarstedt M. Hopkins L. Kuppelwieser V. (2014). Partial least squares structural equation Modelling (PLS-SEM) an emerging tool in business research. Eur. Bus. Rev. 26, 106–121. doi: 10.1108/EBR-10-2013-0128

[ref25] Hair J. F. Hult G. T. M. Ringle C. M. Sarstedt M. (2017). A Primer on Partial Least Squares Structural Equation Modeling. 2nd Edn. Thousand Oakes, CA: Sage.

[ref26] Hair J. F. Black W. C. Babin B. J. Anderson R. E. (2010). Multivariate Data Analysis. 7th Edn. Upper Saddle River, NJ: Prentice Hall.

[ref27] Hayran C. Anik L. Gürhan-Canli Z. (2020). A threat to loyalty: fear of missing out (FOMO) leads to reluctance to repeat current experiences. PLoS One 15:e0232318. doi: 10.1371/journal.pone.0232318, PMID: 32353059 PMC7192437

[ref28] Ho S. Y. Lim K. H. (2018). Nudging moods to induce unplanned purchases in imperfect mobile personalization contexts. MIS Q. 42, 757–778. doi: 10.25300/MISQ/2018/14083

[ref29] Hodkinson C. (2019). Fear of missing Out’(FOMO) marketing appeals: A conceptual model. J. Mark. Commun. 25, 65–88. doi: 10.1080/13527266.2016.1234504

[ref30] Huang X. Cai R. (2021). Does product semantics matter in stimulating impulse buying behavior for internet products? Front. Psychol. 12:676086. doi: 10.3389/fpsyg.2021.676086, PMID: 34497555 PMC8419358

[ref31] Islam T. Pitafi A. H. Arya V. Wang Y. Akhtar N. Mubarik S.. (2021). Panic buying in the COVID-19 pandemic: A multi-country examination. J. Retail. Consum. Serv. 59:102357. doi: 10.1016/j.jretconser.2020.102357

[ref32] Joensuu-Salo S. Sorama K. Viljamaa A. Varamäki E. (2018). Firm performance among internationalized SMEs: The interplay of market orientation, marketing capability and digitalization. Admin. Sci. 8:31. doi: 10.3390/admsci8030031

[ref33] Kamboj S. Sarmah B. Gupta S. Dwivedi Y. (2018). Examining branding cocreation in brand communities on social media: applying the paradigm of stimulus-organism-response. Int. J. Inf. Manag. 39, 169–185. doi: 10.1016/j.ijinfomgt.2017.12.001

[ref34] Kaur K. (2020). Impact of the first phase of movement control order during the COVID-19 pandemic in Malaysia on purchasing behavior of Malaysian consumers Kamaljeet Kaur1*, Mageswari Kunasegaran2, Jaspal Singh3, Selvi Salome4, and Sukjeet Kaur Sandhu5. Horizon 2, 131–144. doi: 10.37534/bp.jhssr.2020.v2.nS.id1038.p131

[ref35] Ku H. H. Kuo C. C. Yang Y. T. Chung T. S. (2013). Decision-contextual and individual influences on scarcity effects. Eur. J. Mark. 47, 1314–1332. doi: 10.1108/03090561311324345

[ref36] Labad J. González-Rodríguez A. Cobo J. Puntí J. Farré J. M. (2021). A systematic review and realist synthesis on toilet paper hoarding: COVID or not COVID, that is the question. PeerJ. 9:e10771. doi: 10.7717/peerj.10771, PMID: 33575133 PMC7849510

[ref37] Lee H. J. Yun Z. S. (2015). Consumers’ perceptions of organic food attributes and cognitive and affective attitudes as determinants of their purchase intentions toward organic food. Food Qual. Prefer. 39, 259–267. doi: 10.1016/j.foodqual.2014.06.002

[ref38] Li C. Wang Y. Lv X. Li H. (2021). To buy or not to buy? The effect of time scarcity and travel experience on tourists’ impulse buying. Ann. Tour. Res. 86:103083. doi: 10.1016/j.annals.2020.103083, PMID: 35501887

[ref39] Li M. Zhao T. Huang E. Li J. (2020). How does a public health emergency motivate people’s impulsive consumption? An empirical study during the COVID-19 outbreak in China. Int. J. Environ. Res. Public Health 17:5019. doi: 10.3390/ijerph17145019, PMID: 32668635 PMC7400470

[ref40] Li X. Y. Wang J. Zhang R. X. Chen L. He C. K. Wang C. Y.. (2020). Psychological status Among anesthesiologists and operating room nurses During the outbreak period of COVID-19 in Wuhan, China. Front. Psychiatry 11:574143. doi: 10.3389/fpsyt.2020.574143, PMID: 33343417 PMC7744586

[ref41] Lim P. L. Yazdanifard R. (2015). What internal and external factors influence impulsive buying behavior in online shopping? Global J. Manage. Bus. Rep. 15, 25–32.

[ref42] Lins S. Aquino S. (2020). Development and initial psychometric properties of a panic buying scale during COVID-19 pandemic. Heliyon 6:e04746. doi: 10.1016/j.heliyon.2020.e04746, PMID: 32895636 PMC7467094

[ref43] Mainolfi G. (2020). Exploring materialistic bandwagon behaviour in online fashion consumption: A survey of Chinese luxury consumers. J. Bus. Res. 120, 286–293. doi: 10.1016/j.jbusres.2019.11.038

[ref44] Martin-Neuninger R. Ruby M. B. (2020). What does food retail research tell us about the implications of coronavirus (COVID-19) for grocery purchasing habits? Front. Psychol. 11:1448. doi: 10.3389/fpsyg.2020.01448, PMID: 32581987 PMC7292029

[ref45] Maxwell A. (2014). Bandwagon effect and network externalities in market demand. Asian J. Manage. Res. 4, 527–532.

[ref46] Mehrabian A. Russell J. A. (1974). An Approach to Environmental Psychology. United States: MIT Press.

[ref47] Mertens G. Gerritsen L. Duijndam S. Salemink E. Engelhard I. M. (2020). Fear of the coronavirus (COVID-19): predictors in an online study conducted in march 2020. J. Anxiety Disord. 74:102258. doi: 10.1016/j.janxdis.2020.102258, PMID: 32569905 PMC7286280

[ref400] Moon M. A. Farooq A. Kiran M. (2017). Social shopping motivations of impulsive and compulsive buying behaviors. UW Journal of Management Sciences 1, 15–27.

[ref48] Park K. Ha J. Park J. Y. (2017). An experimental investigation on the determinants of online hotel booking intention. J. Hosp. Mark. Manag. 26, 627–643. doi: 10.1080/19368623.2017.1284631

[ref49] Pradhan V. (2016). Study on impulsive buying behavior among consumers in supermarket in Kathmandu Valley. J. Bus. Soc. Sci. Res. 1, 215–233. doi: 10.3126/jbssr.v1i2.20926

[ref50] Primanto A. B. Rahmawati R. (2021). The antecedents of impulse buying behavior During Covid-19 pandemic: revealing the role of panic buying, government stimulus, perceived scarcity, and fear appeals. J. Theor. App. Manage. 14, 230–247. doi: 10.20473/jmtt.v14i3.29886

[ref51] Rahi S. (2017). Research design and methods: A systematic review of research paradigms, sampling issues and instruments development. Int. J. Eco. Manage. Sci. 6, 1–5. doi: 10.4172/2162-6359.1000403

[ref52] Rasoolimanesh S. M. Wang M. Roldán J. L. Kunasekaran P. (2021). Are we in right path for mediation analysis? Reviewing the literature and proposing robust guidelines. J. Hosp. Tour. Manag. 48, 395–405. doi: 10.1016/j.jhtm.2021.07.013

[ref53] Rodrigues R. I. Lopes P. Varela M. (2021). Factors affecting impulse buying behavior of consumers. Front. Psychol. 12:697080. doi: 10.3389/fpsyg.2021.697080, PMID: 34149580 PMC8206473

[ref54] Schoenherr T. Ellram L. M. Tate W. L. (2015). A note on the use of survey research firms to enable empirical data collection. J. Bus. Logist. 36, 288–300. doi: 10.1111/jbl.12092

[ref55] Sharma P. Roy R. (2016). Looking beyond first-person effects (FPEs) in the influence of scarcity appeals in advertising: A replication and extension of Eisend (2008). J. Advert. 45, 78–84. doi: 10.1080/00913367.2015.1093438

[ref56] Shayan S. Aneela M. Akram M. S. Ronika C. (2017). Do luxury brands successfully entice consumers? The role of bandwagon effect. Int. Mark. Rev. 34, 498–513. doi: 10.1108/IMR-09-2014-0302

[ref57] Šrol J. Ballová Mikušková E. Čavojová V. (2021). When we are worried, what are we thinking? Anxiety, lack of control, and conspiracy beliefs amidst the COVID-19 pandemic. Appl. Cogn. Psychol. 35, 720–729. doi: 10.1002/acp.3798, PMID: 33821088 PMC8013184

[ref58] Sun S. Xie Z. Yu K. Jiang B. Zheng S. Pan X. (2021). COVID-19 and healthcare system in China: challenges and progression for a sustainable future. Glob. Health 17, 1–8. doi: 10.1186/s12992-021-00665-9 PMC7819629 33478558

[ref59] Swennen G. R. Pottel L. Haers P. E. (2020). Custom-made 3D-printed face masks in case of pandemic crisis situations with a lack of commercially available FFP2/3 masks. Int. J. Oral Maxillofac. Surg. 49, 673–677. doi: 10.1016/j.ijom.2020.03.015, PMID: 32265088 PMC7132499

[ref60] Taylor S. (2021). Understanding and managing pandemic-related panic buying. J. Anxiety Disorders 78:102364. doi: 10.1016/j.janxdis.2021.102364, PMID: 33517219

[ref61] Tynan C. McKechnie S. Chhuon C. (2010). Co-creating value for luxury brands. J. Bus. Res. 63, 1156–1163. doi: 10.1016/j.jbusres.2009.10.012

[ref62] Van Herpen E. Pieters R. Zeelenberg M. (2009). When demand accelerates demand: trailing the bandwagon. J. Cons. Psychol. 19, 302–312. doi: 10.1016/j.jcps.2009.01.001

[ref63] Wu W. Y. Lu H. Y. Wu Y. Y. Fu C. S. (2012). The effects of product scarcity and consumers’ need for uniqueness on purchase intention. Int. J. Cons. Stu. 36, 263–274. doi: 10.1111/j.1470-6431.2011.01000.x

[ref64] Wu Y. L. Li E. Y. (2018). Marketing mix, customer value, and customer loyalty in social commerce: a stimulus-organism-response perspective. Int. Res. 28, 74–104. doi: 10.1108/IntR-08-2016-0250

[ref65] Yang J. Peng M. Y. P. Wong S. Chong W. (2021). How E-learning environmental stimuli influence determinates of learning engagement in the context of COVID-19? SOR model perspective. Front. Psychol. 12:584976. doi: 10.3389/fpsyg.2021.584976, PMID: 33868072 PMC8044515

[ref66] Yuen K. F. Wang X. Ma F. Li K. X. (2020). The psychological causes of panic buying following a health crisis. Int. J. Environ. Res. Pub. Health 17:3513. doi: 10.3390/ijerph17103513, PMID: 32443427 PMC7277661

[ref67] Zhang J. Jiang N. Turner J. J. Pahlevan Sharif S. (2021). The Impact of Scarcity of Medical Protective Products on Chinese Consumers’ Impulsive Purchasing during the COVID-19 Epidemic in China. Sustainability 13:9749. doi: 10.3390/su13179749

[ref68] Zhang W. Leng X. Liu S. (2020). Research on mobile impulse purchase intention in the perspective of system users during COVID-19. Personal Ubiquitous Comp. 1–9. doi: 10.1007/s00779-020-01460-w PMC7486589 32952494

[ref69] Zhang Z. Jiménez F. R. Cicala J. E. (2020). Fear of missing out scale: a self-concept perspective. Psychol. Market. 37, 1619–1634. doi: 10.1002/mar.21406

[ref71] Zheng X. Men J. Yang F. Gong X. (2019). Understanding impulse buying in mobile commerce: An investigation into hedonic and utilitarian browsing. Int. J. Info. Manage. 48, 151–160. doi: 10.1016/j.ijinfomgt.2019.02.010

